# Electroacupuncture for pressure ulcer: a study protocol for a randomized controlled pilot trial

**DOI:** 10.1186/1745-6215-15-7

**Published:** 2014-01-06

**Authors:** Qin-hong Zhang, Jin-huan Yue, Zhong-ren Sun

**Affiliations:** 1 Department of Acupuncture and Moxibustion, First Affiliated Hospital of Heilongjiang University of Chinese Medicine, Harbin 150040, China; 2 Department of Acupuncture and Moxibustion, Second Affiliated Hospital of Heilongjiang, University of Chinese Medicine, 24 Heiping Road, Xiangfang District, Harbin Heilongjiang Province 150040, China

**Keywords:** Electroacupuncture, Pressure ulcers, Bedsores, Randomized controlled trial

## Abstract

**Background:**

Pressure ulcers are one of the most common health complaints, which often take months or years to heal, and affect patients’ morbidity and quality of life. Medical options for pressure ulcers are limited. Electroacupuncture (EA) has been employed to relieve the symptoms for patients with pressure ulcers, but there is limited clinical evidence for its effectiveness.

**Methods/Design:**

This study consists of a randomized controlled trial (RCT) with two parallel arms: a control group and an EA group. Both groups will receive standard wound care (including changing position, using mattresses and cushions, and a good diet) of five sessions per week for a total of 40 sessions during the 8-week treatment period. In addition, the EA group will receive the EA intervention. The following outcome measurements will be used in examination of participants: wound surface area (WSA), visual analogue scale (VAS), and the proportion of ulcers healed within trial period (PUHTP). All the outcomes will be evaluated at the start of the study, at the end of the fourth week, at 8 weeks after randomization, and 1 month after treatment cessation.

**Discussion:**

The aim of this study is to evaluate the effectiveness of EA for the treatment of patients with pressure ulcers.

**Trial registration:**

Chinese Clinical Trial Register: ChiCTR-TRC-11001693

## Background

Pressure ulcers, also known as bedsores or pressure sores, are regions of localized damage to the skin and deeper tissue layers affecting muscle, tendon, and bone [1-3]. These pressure ulcers are caused by unrelieved pressure or pressure in combination with shear and usually over bony prominences, such as the sacrum (tailbone), back, buttocks, heels, back of the head, and elbows [4,5]. Open ulcers can become a source of pain, disability, and infection unless they are adequately treated.

The prevalence and incidence of pressure ulcers differ according to the method of data collection and classification. For example, this type of ulcer is widespread, with prevalence rates varying from 8.8% to 53.2% [6,7] and incidence rates fluctuating from 7% to 71.6% [8,9]. Prevalence rates vary widely depending on patients’ predisposing risk factors, such as poor nutrition, confinement to a bed or wheelchair, and so on [5,10,11]. Pressure ulcers are generally graded I, II, III, and IV, according to the European Pressure Ulcer Advisory Panel and the National Pressure Ulcer Advisory Panel (EPUAP/NPUAP) [12].

Intervention strategies for pressure ulcers often represent a great financial burden on healthcare systems. For example, the annual cost associated with treatment of pressure ulcers has been estimated to range from £1.4 to 2.1 billion in the UK; broadly equal to the total cost of community health services [1,13].

Effective and adequate intervention is an important issue for patients, practitioners, and policy makers. Herbal medicine, acupuncture, and moxibustion are three effective traditional Chinese medicine (TCM) therapies and have been administered for treatment of pressure ulcers in China [1]. However, the current level of evidence is poor because of small sample size, poor quality, rare follow-up, and variation in the composition of acupuncture. Considering these methodological flaws, we will conduct a trial to assess the efficacy of electroacupuncture (EA) for pressure ulcers and the feasibility of a large clinical trial.

## Methods/Design

### Objective

The primary objective of this study is to evaluate the efficacy of EA treatment for pressure ulcers.

### Design

**Figure 1:**
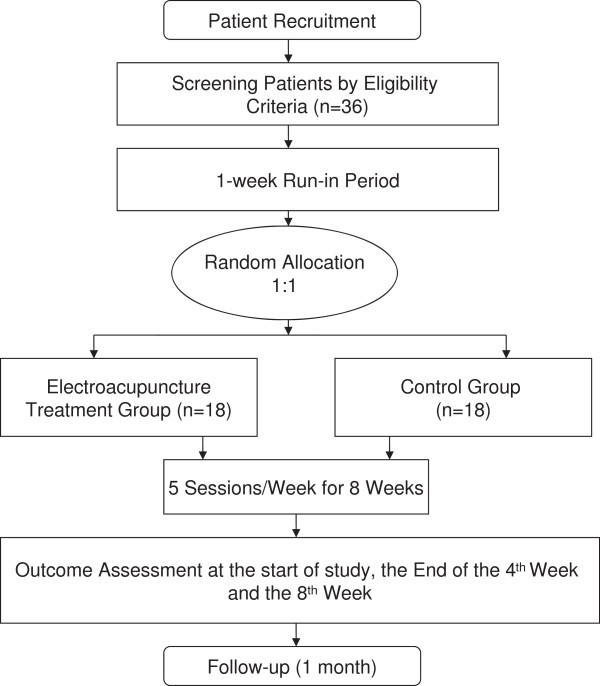
Trial flow chart.

This study will consist of a randomized, assessor- and analyst-blinded, controlled trial to compare an EA group with a control group (Figure 1 and Table 1). The trial will be conducted at the Second Affiliated Hospital of Heilongjiang University of Chinese Medicine, Heilongjiang Province, China.

**Table 1:** Visit times and data collection

**Data collection**	**-1 week**	**0 week**	**4 weeks**	**8 weeks**	**1 month after treatment phase**
	**Baseline**	**Treatment phase**			**Follow-up phase**
**Patients**					
Informed consent	**×**				
Sign the informed consent		**×**			
Medical history	**×**				
Physical examination	**×**				
Randomization		**×**			
**Intervention**					
EA group (n = 18)		40 sessions of EA; plus standard wound care			
**Comparison**					
Control group (n = 18)		40 sessions of standard wound care			
**Outcomes**					
WSA		**×**	**×**	**×**	**×**
VAS		**×**	**×**	**×**	**×**
PUHTP		**×**	**×**	**×**	**×**
**Safety**					
Adverse events		**×**	**×**	**×**	**×**

EA, electroacupuncture; PUHTP, proportion of ulcers healed within trial period; VAS, visual analogue scale; WSA, wound surface area; the symbol ‘x’ is used as a check mark.

This trial will include an 8-week treatment period and a 1-month follow-up period. After randomization, patients will receive a total of 40 session treatments over a period of 8 weeks. Outcome measurements will be assessed at baseline (1 week after participants are diagnosed with pressure ulcer), as well as at the end of the fourth week and 8 weeks after randomization, and 1 month after conclusion of the treatment phase. Patients will be informed that they may be assigned to an EA group, or control group.

This trial will be carried out according to the principles of the Declaration of Helsinki (version Seoul, 2008). This study protocol has been approved by the ethical review boards of the Second Affiliated Hospital of Heilongjiang University of Chinese Medicine, with permission number LLP2011012. All participants will be asked to provide written informed consent before enrollment, and will be given sufficient time to reach a decision to participate and sign the consent form.

### Randomization

A randomization scheme will be performed at the Good Clinical Practice (GCP) Center of the Second Affiliated Hospital of Heilongjiang University of Chinese Medicine. This scheme will be used to assign participants to the EA group and control group in a 1:1 ratio. The randomization scheme will be generated by the central computer system. Two separate databases will be set up: the ‘participants’ database that includes basic participant information, such as name, contact details, and so on; and the ‘randomization’ database that holds data of patients registered on the trial and their allocation [14]. The practitioners participating in the study will not take part in the randomization process. Patients who meet the inclusion criteria and agree to provide written informed consent will be included in the study. The researcher will then contact the GCP center where the participant’s information is registered, and the randomization information of the patient’s assignation to one of the two study branches will be sent to the researcher by cell phone or via email. This procedure will ensure adequate randomization concealment, and is not influenced by the researcher taking part in this study.

### Blinding

The researchers are in charge of sending randomization information and also receiving notices of the patient’s assignation. It is not practical to blind the researchers to treatment allocations. In addition, it is not possible to prevent participants from knowing if they have received EA treatment or standard wound care. However, it is feasible to blind outcome assessors and statisticians.

### Recruitment

Participants will be recruited via advertisements and posted notices. Advertisements will be broadcast on local television channels and also published in local newspapers. Posted notices will be put up on the notice board of the local hospital. A data compilation form including all variables of interest and all potential risks will be completed by the corresponding research center. The obtained information will be recorded on an electronic database for subsequent statistical analysis.

### Eligibility

#### Inclusion criteria

Participants will be included if they fulfill the following criteria: 1) pressure ulcers of grade II or III, according to EPUAP/NPUAP [12]; 2) duration of one or more pressure ulcers is greater than 3 months; and 3) aged 18 to 75 years old.

#### Exclusion criteria

Patients with any of the following conditions will be excluded: 1) undergoing any other therapies which might interfere with the ability to heal, such as corticosteroid therapy, radiation therapy, or chemotherapy for cancer; 2) severe diseases, such as severe liver, cardiac, kidney diseases, and relevant intensive complications, for reasons of safety as well as evaluation of pain intensity for patients with sensation loss; and 3) medical conditions for which EA is contraindicated [15], such as ventricular arrhythmia, atrial fibrillation, using cardiac pacemaker, history of deep radiation therapy within the local region, known deep venous thrombosis or thrombophlebitis, superficial metal ions or metal implants near the area, pregnancy, or active osteomyelitis.

### Intervention

The intervention was designed according to the record in the ancient book of *Yellow Emperor’s Inner Bible,* in Chapter *Lingshu*-*Guanzhen*[16] and according to the recent study of acupuncture as a treatment for pressure ulcers [17]. Standard wound care is a non-EA therapy option in this trial. The EA protocol was developed in consensus with acupuncturists and experts, who are good at acupuncture. In addition, this protocol is consistent with the Standards for Reporting Interventions in Clinical Trials of Acupuncture (STRICTA) guidelines for the performance of EA studies [18].

### Control group

Standard wound care will be used in the control group. In this study, standard wound care will include moving around and changing position, using mattresses and cushions, and a good diet. Keeping moving by moving around and changing position as much as possible should be restricted to less than 2 hours. Using mattresses and cushions can reduce the pressure on bony parts of the body. For patients with pressure ulcers of grade II, they should use a high specification foam mattress. As for pressure ulcers of grade III, patients should receive a more sophisticated mattress or overlay. A good diet of eating well and drinking enough water is very important. The healthcare professional will offer good dietary advice according to what is patients missing from the diet, their general health, and preferences.

### Treatment group

In addition to standard wound care, all participants in the treatment group will receive EA treatment. Hanyi needles (0.17 × 7 mm; Tianjin Medical Appliance Factory, Tianjin, China) and a Micro Plus transcutaneous electrical nerve stimulator (BioMedical Life Systems, Inc, Vista, CA, USA) will be used in the trial. Two needles will be punctured into the skin of the local wound. One needle will be inserted into the wound center with a 90° angle and connected with the negative pole, while the other needle will be punctured in the normal skin 0.5 cm away from the ulcer margin with a 45° angle and connected with the positive pole. Both needles will be applied without lifting, thrusting, or rotating. The electric stimulator will be turned on with 500 μA, 0.5 Hz, 30 minutes each time, for five sessions per week for 8 weeks.

### Outcome measures

#### Primary outcome

1) Wound surface area (WSA)

The pressure ulcer surface area will be measured by the use of acetate tracing and subsequent planimetric determination. Ulcer tracings will be accomplished by outlining the pressure ulcer circumference onto a transparent film applied directly over the wound. Each ulcer will be traced three times by two assessors, respectively, in order to improve the accuracy of the tracings. The ulcer surface area will be determined from the wound tracing using a planimeter (KP-21C) by a third assessor. All three assessors will be blinded as to the identity of the patient and to the treatment group assignment.

#### Secondary outcome

1) Visual analogue scale (VAS)

The pain intensity of pressure ulcers will be assessed using the 10 cm VAS (0, absence of pain; 10, worst pain imaginable) [19,20]. The VAS was selected as a secondary outcome measurement in order to evaluate the clinical severity and impact on activities of daily life in patients with pressure ulcers.

2) Proportion of ulcers healed within trial period (PUHTP)

The complete healed ulcers were defined as 100% epithelization or skin closure without drainage.

### Statistical methods

#### Sample size

This study is a pilot study for the evaluation of the efficacy of EA for patients with pressure ulcers and the feasibility of a large clinical trial. Because of the short duration, lasting 8 weeks, the desired sample size for this pilot study is 36 participants, with 18 participants in each group, assuming a dropout rate of 20%, which is the minimum sample size necessary to evaluate the effect of EA [21].

#### Analysis

Data will be analyzed by a statistician blinded to the allocation of groups. Statistical analyses will be conducted using the SPSS 17.0 statistical software packages (IBM, Armonk, NY, USA), and the levels of significance will be reported at *P* <0.05. The intention-to-treat (ITT) population will be defined as the participants who are randomized and received at least one treatment session. The data analysis of baseline characteristics, as well as the primary and secondary outcomes will be based on the ITT principle. Analysis of covariance will be conducted unless the adjustment for possible baseline incomparability is needed.

#### Data handling

Investigators will enter the collected data required by the protocol into the case report forms. Non-obvious errors or omissions will be recorded on data query forms, which will be returned to the researchers’ workshop for resolution. The data from all centers will be pooled and summarized with respect to demographic baseline characteristics, effectiveness, and safety observations.

#### Patient safety

Any adverse experiences (known as unfavorable or unintended signs, symptoms, or diseases occurring after treatment) related to EA treatment will be monitored. The research team will review all trial protocols, monitor participant safety, and investigate any adverse events. The trials will be terminated if there are concerns about patient safety.

#### Quality control

All staff will be required to undergo special training, including patient selection and exclusion, completing the case report form, and acupuncture method, before participating in the trial. Monitors will check case report forms and EA operation at the participating hospital once a month. Drop-outs and withdrawals (and the reasons) from the study will be fully documented throughout the treatment and follow-up periods.

### Ethics

Written informed consent will be obtained from each participant. This study is approved by the ethical review boards of the Second Affiliated Hospital of Heilongjiang University of Traditional Chinese Medicine.

## Discussion

Pressure ulcers remain a major public health problem. The results of this study will determine if EA is an effective intervention for patients with pressure ulcers. The results will also identify whether this therapy focuses on symptomatic relief.

Acupuncture, a kind of TCM, has been a form of healthcare in China for thousands of years. It has been reported to be of some benefit to patients with pressure ulcers, and could also enhance some of their signs and symptoms.

Currently, there are no randomized controlled trials (RCTs) about EA treatment for patients with pressure ulcers; therefore, we designed this study. This study aims to conduct a pilot study for a full-scale trial of EA for pressure ulcers, and to examine its potential effect in terms of pressure ulcers of future episodes. In addition, it also provides the feasibility of a larger clinical trial. The data pooled will shed new light on acupuncture, especially for EA intervention for pressure ulcers.

## Trial status

The trial is currently recruiting participants.

## Abbreviations

ChiCTR: Chinese clinical trial register; EA: Electroacupuncture; EPUAP: European pressure ulcer advisory panel; GCP: Good clinical practice; ITT: Intention-to-treat; NPUAP: National pressure ulcer advisory panel; PUHTP: Proportion of ulcers healed within trial period; RCT: Randomized controlled trial; STRICTA: Standards for reporting interventions in clinical trials of acupuncture; TCM: Traditional Chinese medicine; VAS: Visual analogue scale; WSA: Wound surface area.

## Competing interests

The authors declare that they have no competing interests.
